# The position of the polish bishops’ conference on LGBT+ – philosophical, theological, clinical and political aspects

**DOI:** 10.1192/j.eurpsy.2021.998

**Published:** 2021-08-13

**Authors:** W. Kosmowski

**Affiliations:** Department Of Psychiatry, Collegium Medicum, Nicolaus Copernicus University, Bydgoszcz, Poland

**Keywords:** theology, sexual medicine, ethics

## Abstract

**Introduction:**

Practicing medicine cannot disregard cultural conditions. Philosophy and religion are elements of culture. For several years in Poland, various circles have discussed the extensive LGBT issues. A document of Polish Bishops on this subject appeared on 28.08.2020. In 2018, 91.8% of people over 16 years old in Poland declared affiliation to the Roman Catholic Church (Statistics Poland 2020).

**Objectives:**

The aim of the study is to present different perspectives of effects of that publication, including ethical evaluation and references to clinical practice.

**Methods:**

Statements of protagonists and antagonists of this document in Polish were analyzed. Collected arguments were divided into types: philosophical – by philosophy branches (e.g. ethics, philosophical anthropology), theological and clinical.

**Results:**

As of 29.09.2020 – 85,200 results in the Google Search after typing (in Polish) “Polish Episcopal Conference LGBT”. The use of philosophical arguments by both parties results from the adaptation of different systems, e.g. regarding philosophical anthropology, some assume the immutability of human nature, others – its variability and susceptibility to shaping, e.g. human sexuality. Some emphasize the importance of non-discrimination, while others indicate the need to consider human essence in determining directions of actions.

**Conclusions:**

Professionals should help everyone, regardless of conditions, in accordance with conscience and contemporary medical knowledge [Polish Code of Medical Ethics]. They should try to understand patients and the context of symptoms. Familiarizing oneself with arguments of both sides helps in this. But polemical language makes dialogue difficult. What for one is a “venerable tradition”, for another is a “stereotype” or “discrimination”.

